# Understanding of a Ni‐Rich O3‐Layered Cathode for Sodium‐Ion Batteries: Synthesis Mechanism and Al‐Gradient Doping

**DOI:** 10.1002/smll.202408072

**Published:** 2024-11-08

**Authors:** Binglu Wang, Xiangze Kong, Filipp Obrezkov, Princess Stephanie Llanos, Jani Sainio, Alisa R. Bogdanova, Anna Kobets, Timo Kankaanpää, Tanja Kallio

**Affiliations:** ^1^ Department of Chemistry and Materials Science School of Chemical Engineering Aalto University Kemistintie 1 Espoo 02150 Finland; ^2^ Department of Applied Physics School of Science Aalto University Puumiehenkuja 2 Espoo 02150 Finland; ^3^ Umicore Battery Materials Finland Kokkola 67101 Finland

**Keywords:** ALD, gradient doping, in situ HT‐XRD, NaNi_0.8_Mn_0.1_Co_0.1_O_2_, *operando* XRD, O3‐type cathode, sodium‐ion batteries

## Abstract

O3‐type NaNi_0.8_Mn_0.1_Co_0.1_O_2_ (NaNMC811) cathode active materials for sodium–ion batteries (SIBs), with a theoretical high specific capacity (∼ 187 mAh g^−1^), are in the preliminary exploration stage. This study comprehensively investigates NaNMC811 from multiple perspectives. For the first time, the phase evolution ($P\bar{3}m1$ ‐ $Fm\bar{3}m$ ‐ $R\bar{3}m$) during the solid‐state synthesis is systemically investigated, which elucidates in‐depth the mechanisms of the thermal sodiation process. Furthermore, an Al‐gradient doping of NaNMC811 was successfully implemented through Al_2_O_3_ coating on the cathode active material (CAM) precursor. The modified Al−NaNi_0.8_Mn_0.1_Co_0.1_O_2_ (Al‐NaNMC811) exhibits excellent electrochemical dynamics and performance, maintaining a specific capacity above 100 mAh g^−1^ after 100 cycles at 0.1 C (1.5–4.1 V) while providing a promising capacity retention of 63%. Additionally, the material demonstrates excellent rate capabilities, retaining a specific capacity of 107 mAh g^−1^ at 5 C. Compared to pristine NaNMC811, the modified Al‐NaNMC811 is proven to have improved electrochemical kinetics with a higher Na^+^ diffusion coefficient due to dilated (003) interplanar spacing, and a more stable structure during the electrochemical charge–discharge processes, which is attributed to stronger Al–O bond energy. Understanding phase formations during the synthesis and comprehensive insight in the gradient doping for O3‐type NaNMC811 CAMs guides further development of next‐generation SIBs materials.

## Graphical abstract

To lay the foundations for synthesis, this work investigates the complex phase evolution during solid‐state synthesis of NaNi_0.8_Mn_0.1_Co_0.1_O_2_, a high‐capacity cathode material for sodium‐ion batteries. Innovative Al‐gradient doping improves the electrochemical kinetics and structure stability and enables an 18% capacity retention improvement at a high discharging current and impressive capacity retention after 100 charge–discharge cycles.

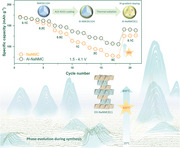

## 1 Introduction

The dominance of lithium‐ion battery (LIB) technology in the energy storage market is primarily driven by its high energy density, long lifetime, and mature manufacturing technology.^[^ ^1^, ^2^, ^3^ ^]^ However, supply fluctuations and uneven distribution of lithium reserves have become crucial issues for the LIBs industry.^[^ ^4^, ^5^, ^6^ ^]^ Thus, there is a growing interest in research and development of alternatives for LIBs. Among post‐LIB technologies, sodium‐ion batteries (SIBs) are emerging as a highly potential alternative due to abundant sodium reserves and low cost of sodium compared to lithium.^[^ ^7^, ^8^, ^9^, ^10^ ^]^

Cathode materials play an essential role in the electrochemical performance of SIBs.^[^ ^11^, ^12^, ^13^, ^14^ ^]^ NaNi_*x*_Mn_*y*_Co_*z*_O_2_ (NaNMC where x+y+z = 1) layered oxide is a SIB cathode material class known for relatively high energy density, facile synthesis method, and cost‐effectiveness.^[^ ^15^, ^16^, ^17^, ^18^, ^19^, ^20^, ^21^, ^22^ ^]^ NaNMC can be categorized into P2,^[^ ^23^, ^24^, ^25^ ^]^ O3,^[^ ^10^, ^26^ ^]^ O2,^[^ ^7^, ^27^ ^]^ and P3^[^ ^12^, ^28^ ^]^ types based on sodium coordination and varied layered structures. Among these, the O3 types are the most extensively studied due to their high specific capacity, especially when Ni content is high (x ⩾ 0.6).^[^ ^10^, ^13^, ^29^ ^]^ In the O3‐type NaNMC, Na ions are located between TMO_6_ (TM = Ni, Mn, and Co) edge‐sharing layers with octahedral coordination. Due to this structural arrangement, higher migration energy barriers are imposed on Na ions caused by the tortuous pathways, involving transfer through tetrahedral vacancies and subsequent migration into octahedral sites, leading to poor diffusion kinetics.^[^ ^13^, ^30^, ^31^ ^]^ Several crystal structure changes, intergranular cracks, and other mechanical damages may occur at the atomic, particle, and electrode level during charge–discharge cycles. Due to these issues, O3‐type cathodes suffer from drastic capacity decreases during the initial cycling period, resulting in low capacity retention.^[^ ^15^, ^17^, ^22^, ^32^ ^]^ To address the aforementioned issues, surface coating and bulk doping are investigated for O3‐type layered cathode active materials.^[^ ^17^, ^18^, ^19^, ^32^, ^33^ ^]^

Hwang et al. reported that Al_2_O_3_‐coated NaNi_0.6_Mn_0.2_Co_0.2_O_2_ exhibits a high specific capacity (151 mAh g^−1^) and 91% capacity retention after 50 cycles in half cells.^[^ ^19^ ^]^ Similarly, MgO, ZrO_2_, and TiO_2_ coatings have also been explored for SIB cathodes.^[^ ^34^, ^35^ ^]^ These metal oxide coatings protect the NaNMC surface and suppress cathode‐electrolyte interface (CEI) formation.^[^ ^19^, ^34^ ^]^ On the other hand, bulk doping enhances ionic conductivity, mechanical strength, and structure stability.^[^ ^17^, ^18^, ^19^ ^]^ Al^3 +^ is a popular dopant for O3‐type layered oxides^[^ ^26^, ^32^, ^36^, ^37^ ^]^ substituting transition metal (TM) in the layered structure.^[^ ^26^ ^]^ Doping with Al^3 +^ is suggested to enhance structural stability because of the higher bond dissociation energy of Al–O (502 kJ mol^−1^) compared to Ni–O (366 kJ mol^−1^), Mn–O (402kJ mol^−1^), and Co‐O (368 kJ mol^−1^).^[^ ^38^ ^]^ In particular, Kumar and his co‐workers proposed an Al^3 +^ doping of NaNi_0.5_Mn_0.3_Co_0.2_O_2_, which yielded in a material maintaining 90% of its original capacity (120 mAh g^−1^) after 200 cycles.^[^ ^26^ ^]^ However, doping is not able to address both surface and bulk degradation simultaneously. The use of gradient doping, an approach producing a dopant‐rich surface and moderate bulk doping,^[^ ^39^ ^]^ has successfully addressed this concern in layered oxide LIB cathode materials.^[^ ^36^, ^40^ ^]^ However, such an innovative modification method remains unexplored for SIB layered oxide cathode materials.

Although pristine and modified O3‐type NaNMCs have been investigated, understanding of Ni‐rich NaNi_0.8_Mn_0.1_Co_0.1_O_2_ (NaNMC811) with attractive specific capacity (∼ 187 mAh g^−1^) is still preliminary.^[^ ^15^ ^]^ There is an urgent need to investigate the fundamental properties and electrochemical mechanisms of NaNMC811, to provide alternatives to commercial cathodes currently available. Specifically, it is essential to investigate the phase formations during synthesis of NaNMC811 to select the suitable sodiation conditions when using a solid‐state synthesis method. However, to the best of our knowledge, no investigations have been carried out on the complexity of the sodiation process for NaNMC811 so far.

This work provides a comprehensive investigation of the structural, morphological, and electrochemical performance of a pristine and gradient doping modified O3‐type NaNMC811 cathode material for SIBs. in situ high‐temperature X‐ray diffraction (in situ HT‐XRD) is employed to investigate the phase transition mechanisms occurring in NaNMC811 during solid‐state synthesis. Using atomic layer deposition (ALD) technology, a thin uniform layer is coated on an industrial precursor, which turns into Al‐gradient doped NaNMC811 (Al‐NaNMC811) with a low Al amount (0.16 wt%) after thermal sodiation. Such a small dopant amount of electrochemically inert Al is deemed beneficial and expected to improve structural stability without decreasing the capacity. From the electrochemical investigations, the Al modification is shown to successfully improve the electrochemical kinetics and consequently, the electrochemical performance. especially the rate capability. *Operando* X‐ray diffraction (*operando* XRD) reveals that the Al‐NaNMC811 structure is more stable than that of the pristine NaNMC811. This study fills a knowledge gap on the gradient doping of SIB cathode materials. The fundamental knowledge of Ni‐rich NaNMC811 synthesis and modification provided in this study can guide future studies focusing on achieving excellent electrochemical performance for SIBs.

## 2 Result and Discussion

### 2.1 Phase Formation Mechanism

**Figure 1:**
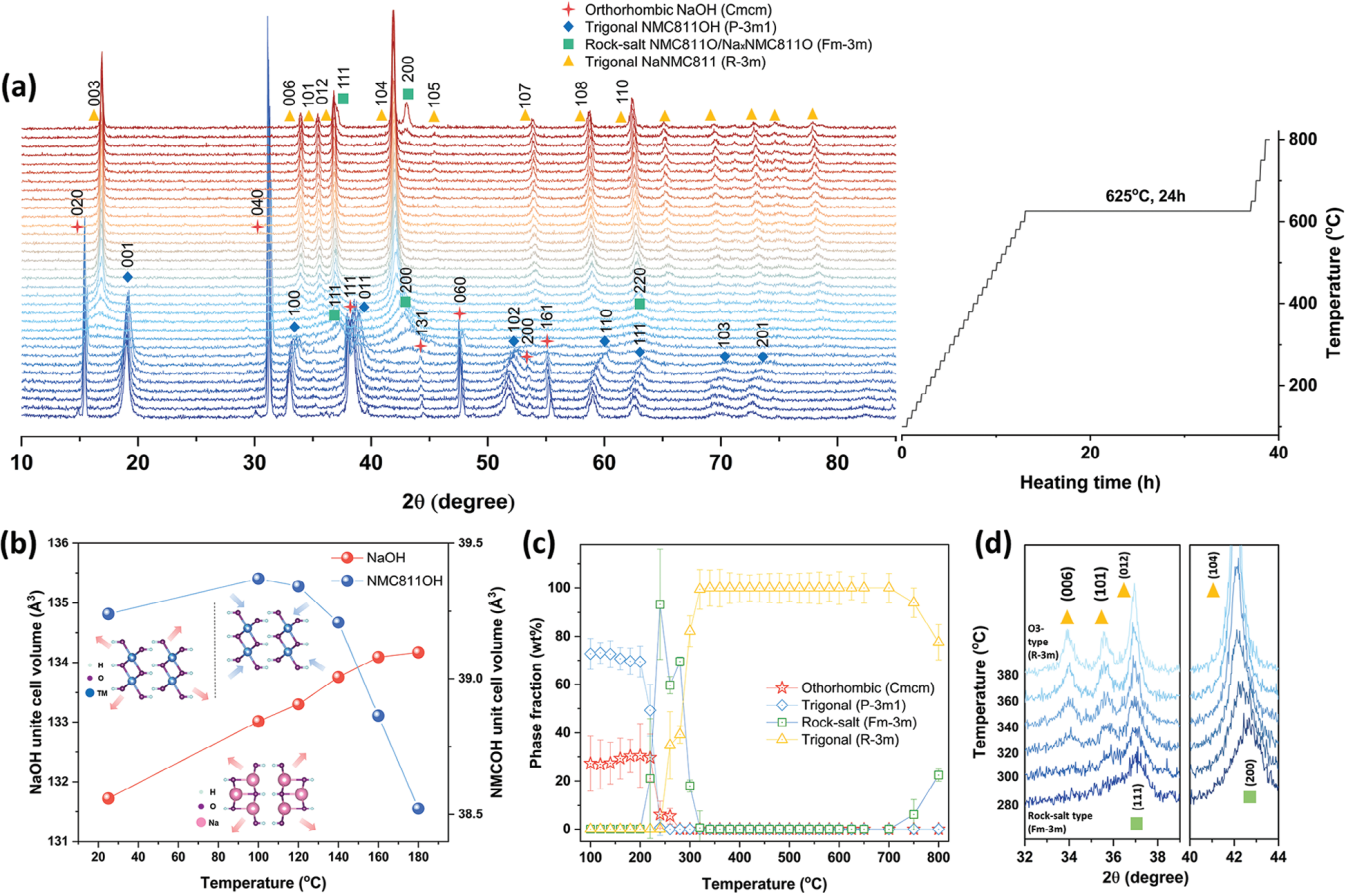
Analysis of the phase formation mechanism via in situ HT‐XRD. a) in situ HT‐XRD patterns of the synthesis process for NaNMC811, heating from 25 °C to 800 °C under the ambient conditions, b) unit cell volume change of NaOH and NMC811OH as a function of heating temperature, c) the corresponding phase fraction evolution of the NaOH and NMC811OH mixture as a function of heating temperature, d) transition from the rock‐salt phase into trigonal layered phase.

For SIB layered oxide cathodes, the synthesis temperature typically is lower than those utilized for similar LIB active materials with equivalent Ni contents.^[^ ^15^, ^18^, ^41^, ^42^ ^]^ A higher Ni content tends to increase the sensitivity of Ni_0.8_Mn_0.1_Co_0.1_(OH)_2_ (NMC811OH) precursors to temperature variations, potentially resulting in a more complex behavior during the calcination process.^[^ ^15^, ^43^ ^]^ To investigate the phase formation mechanism of NaNMC811 during solid‐state synthesis, in situ HT‐XRD was carried out from 25 °C to 800 °C, during which a series of phase changes occur, as depicted in **Figure** **1**a. The in situ HT‐XRD patterns were also measured after the synthesis period, specifically when the sample was cooled down to room temperature (Figure S1, Supporting Information). The XRD patterns indicate that the phase formed at high temperatures remains unchanged during cooling.

During sodiation, as the temperature ramps up from room temperature to 200 °C, a slight shift in the peak positions of NaOH (to lower 2θ angle) and NMC811OH (to higher 2θ angle) in the starting material mixture is observed (Figure S2a, Supporting Information). These are attributed to thermal expansion and subsequent H/O loss, respectively. No new peaks emerge when the temperature increases up to 200 °C. Subsequently, distinct phase transition processes appear between 200 °C and 320 °C, indicating the onset of NMC811OH decomposition. This results to the generation of a rock‐salt type oxide Ni_0.8_Mn_0.1_Co_0.1_O (NMC811O), accompanied by the disappearance of the peaks which belong to NaOH (Figure S2b, Supporting Information).^[^ ^43^ ^]^ Starting at 320 °C, all peaks related to NaNMC811 are formed, exhibiting a single‐phase structure, which continuously grows until 625 °C. However, after the temperature rises above 700 °C, an impurity peak between 43°‐ 44° appears, which has been designated to NiO (PDF#47‐1049) (Figure S2c, Supporting Information) in a previous study.^[^ ^44^ ^]^

To quantitatively investigate the phase transition process, the XRD patterns were analyzed by Rietveld refinement and the results are shown in Figure S3 (Supporting Information). As depicted in Figure 1b, NaOH initially shows a thermal volume expansion from room temperature (131.768 Å^3^) until 180 °C (134.132 Å^3^). Meanwhile, NMC811OH expands from room temperature (39.240 Å^3^) to 100 °C (39.369 Å^3^), and then contracts until 180 °C (38.522 Å^3^). This unusual “heat contraction” is ascribed to decreases in the distance of H atoms that bond to different O atoms. The phenomenon is plausibly related to structural changes leading to the next reaction stage during which NMC811OH loses water. Afterwards, the following reaction proceeds in the range of 200 °C–320 °C:

(1) $${\mathrm{Ni}}_{0.8}{\mathrm{Mn}}_{0.1}{\mathrm{Co}}_{0.1}(\mathrm{OH})2\overset{\Delta }{\to }{\mathrm{Ni}}_{0.8}{\mathrm{Mn}}_{0.1}{\mathrm{Co}}_{0.1}O+H_{2}O$$

Reaction 1 displays the hydroxide precursor (space group $P\bar{3}m1$) conversion into a rock‐salt phase ($Fm\bar{3}m$), which occurs below 260 °C. Above 260 °C, the $P\bar{3}m1$ phase completely disappears (Figure 1c). The refinement results indicate that NMC811O has the highest phase fraction under 240 °C (93.26%) with the lattice parameter *a* = *b* = *c* = 4.1978(7) Å and unit cell volume 73.975 Å^3^. The calculated chemical composition is Ni_0.806_Mn_0.1_Co_0.1_O_1_, identical to NMC811O. In contrast, NaOH begins to participate in the reaction when Na^+^ is inserted into the NMC811O crystal structure starting from 220 °C. The resulting structure is a sodium‐deficient [Na_*x*_(Ni_0.8_Mn_0.1_Co_0.1_)_1−*x*_]O (Na_*x*_NMC811O), whereby, the rock‐salt structure stays the same with a constant valence for each element.^[^ ^43^ ^]^

With the continuous insertion of Na^+^ and O_2_ (from ambient air) into the crystal structure, Na^+^ begins to migrate inside the crystals while the transition metals (TMs) rearrange, leading to the formation of the O3‐layered type NaNMC811 ($R\bar{3}m$, Figure 1d).

**Figure 2:**
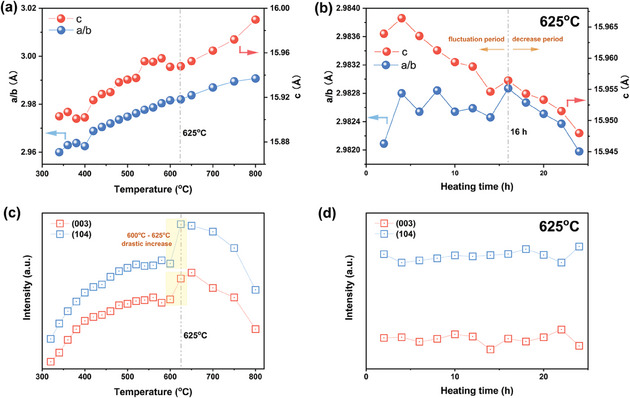
Structural evolution of NaNMC811. Variations of the lattice parameters *a*, *b*, and *c* at a) 320 °C to 800 °C and b) 625 °C thermostatic treatment for 24 h. Absolute intensity change of the (003) and (104) peaks c) from 320 °C to 800 °C and d) 625 °C thermostatic treatment for 24 h.

As the temperature is further elevated from 320 °C to 700 °C, only NaNMC811 exists. In general, the *a*, *b*, and *c* lattice parameters continue to increase, with slight fluctuations (**Figure** **2**a). As the temperature is held at 625 °C, all the parameters tend to fluctuate at first and begin to decrease after 16 h, which is probably associated with the surface reorganization and optimization of the crystal structure (Figure 2b).^[^ ^43^ ^]^ It has been proven for O3‐layered LIBs cathodes that the *R* ratio (*R* = I(003)/I(104)), expected to exceed 1.2, is valuable for analyzing Li and TM cations mixing.^[^ ^45^, ^46^, ^47^ ^]^ However, such cation mixing is absent in NaNMC811 due to a larger size of Na^+^ (102 pm) compared to Ni^2 +^ (69 pm), which is comparable to Li^+^ (76 pm). This enables identifying the crystallinity directly by the intensity of the peaks in the continuous‐pattern‐recording in situ HT‐XRD. As seen in Figure 2c, the intensity of the (003) and (104) peaks steadily increase starting from 320 °C until 600 °C, and after that drastically increase from 600 °C to 625 °C. Moreover, minor changes in the intensity are observed when maintaining the sample for 24 h at 625 °C (Figure 2d), followed by quick decrease over 650 °C. After 700 °C (Figure 1c), the unexpected rock‐salt phase starts to appear and grows rapidly, which can be attributed to O loss and cations migration.

Based on the in situ HT‐XRD results, a temperature range from 600 °C to 625 °C combined with a minimum of 16 h calcination time produce a well‐ordered crystalline NaNMC811 structure, which is expected to have an excellent electrochemical performance. The overall chemical reaction of the synthesis process is described by Reaction 2.

(2) $$\begin{array}{ccc} & & 2{Ni}_{0.8}{\mathrm{Mn}}_{0.1}{\mathrm{Co}}_{0.1}{\left(\mathrm{OH}\right)}_{2}+2\mathrm{NaOH}\\ & & \;+\;\frac{1}{2}O_{2}\overset{\Delta }{\to }2{\mathrm{NaNi}}_{0.8}{\mathrm{Mn}}_{0.1}{\mathrm{Co}}_{0.1}O_{2}+3H_{2}O \end{array}$$

**Figure 3:**
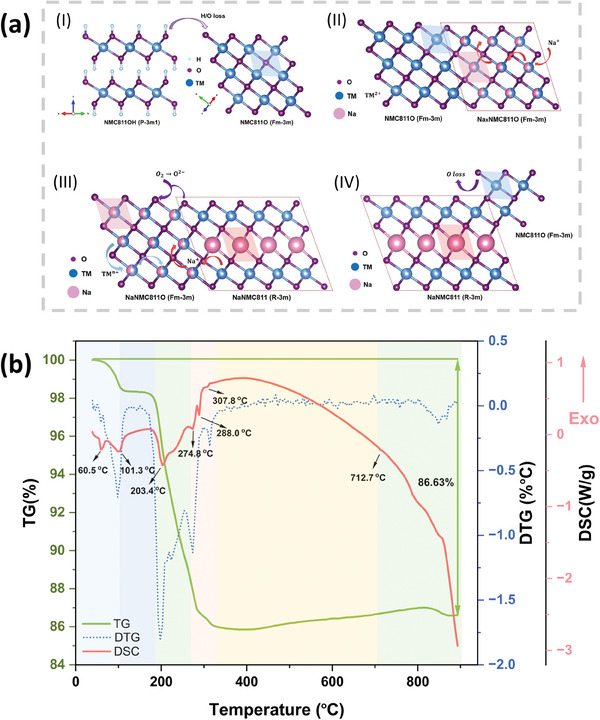
H/O loss from NMC811OH, sodium/oxygen incorporation into the rock‐salt‐type Na_*x*_NMC811O phase, and reformation of impurities above 700 °C. a) Schematic diagram of the phase transition under the heating temperature: (I) dehydration of NMC811OH; (II) Na^+^ insertion into the rock‐salt phase; (III) migration of Na^+^ and O capture; (IV) O loss and reformation of an impure phase. b) TG, DTG and DSC plots for the NaOH and NMC811OH mixture as a function of heating temperature from 40 °C to 900 °C.

**Figure** **3**a provides a schematic representation of the above chemical reaction process with cation migration, using ball‐and‐stick models.^[^ ^43^ ^]^

To confirm the above analysis, thermogravimetric (TG) and differential scanning calorimetric (DSC) experiments were carried out under comparable heating conditions in air, and the resulting TGA‐DSC curve is shown in Figure 3b. Two endothermic peaks under 60.5 °C and 101.3 °C coincide with 1.65% mass loss, which is related to water evaporation from the sample. A drastic mass decrease is observed between 180 °C and 320 °C, proving the H/O loss from the hydroxide NMC811OH compounds as illustrated in Reaction 1. The endothermic peak at 203.4 °C is due to the dehydration of NMC811OH and the following three endothermic peaks (274.8 °C, 288.0 °C, 307.8 °C) may be related to the Na^+^ insertion process, which corresponds to the decreasing phase fraction of NaOH (space group *Cmcm*, Figure 1c) observed by XRD. The O_2_ insertion into the layered structure, synchronously occurring with Na^+^ migration inside the crystalline to form NaNMC811 (Figure 1d), is detectable in the TG measurement as a mass‐increasing region.^[^ ^43^ ^]^ When the temperature increases, the formation of NMC811O observed in in situ HT‐XRD is confirmed by the small endothermic peak at 712.7 °C, which is suggested to be induced by the cations re‐migration. The slight mass decrease signifies the O loss after 820 °C.^[^ ^48^ ^]^

### 2.2 Structure and Morphology Characterizations

**Scheme 1:**
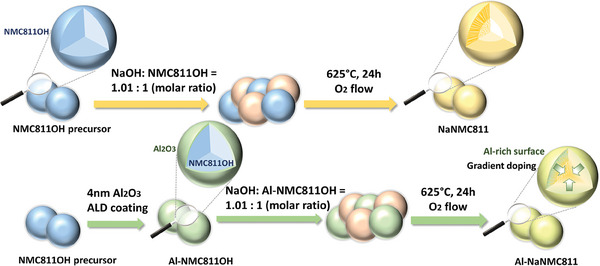
Schematic illustration of the synthesis of NaNMC811 and Al‐NaNMC811.

Based on the conditions identified by in situ HT‐XRD for synthesizing NaNMC811 (*cf*. the discussion in the section “Phase formation mechanism”), a solid‐state synthesis was carried out at 625 °C for 24 h, which successfully produced phase pure samples. As illustrated in **Scheme** 1, an Al_2_O_3_ coating was also applied using ALD technology, which allows precise control of the coating thickness. Al‐gradient doping (0.16 wt%) was achieved subsequently through the thermal sodiation using the same conditions as for the non‐doped material, leading to the final production of Al‐NaNMC811.

**Figure 4:**
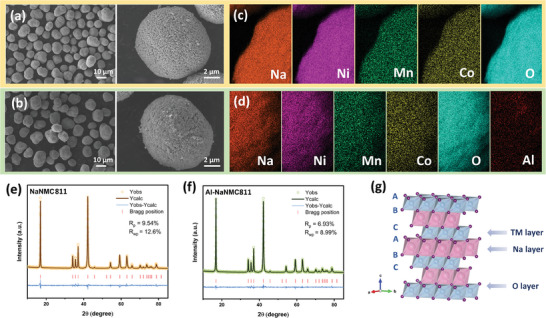
Morphological and structural characterizations of NaNMC811 and Al‐NaNMC811. SEM images of a) NaNMC811 and b) Al‐NaNMC811; EDS mapping images of c) NaNMC811 and d) Al‐NaNMC811; XRD Rietveld refinement results for e) NaNMC811 and f) Al‐NaNMC811, and g) Schematic illustration of the O3‐type crystal structure.

**Figure 5:**
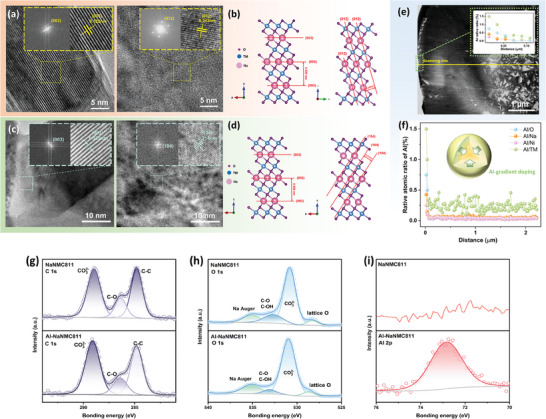
Typical TEM images for a) NaNMC811, c) Al‐NaNMC811. Schematic diagram of typical crystal planes for the O3‐type structure of b) (003) and (012) for NaNMC811, d) (003) and (104) for Al‐NaNMC811. e) Cross section and schematic diagrams of a radial line scan for Al‐NaNMC811, f) linear sweep EDX signals of the Al‐NaNMC811 line scan. High resolution XPS spectra of NaNMC811 and Al‐NaNMC811 for g) C 1s, h) O 1s, and i) Al 2p.

**Figure 6:**
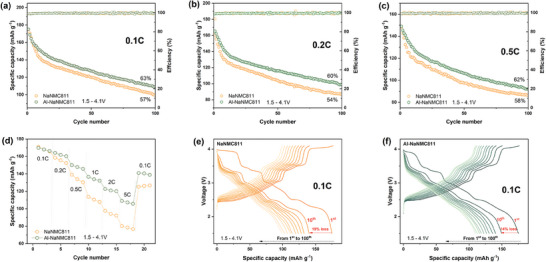
Electrochemical performance at room temperature. Capacity retentions of the NaNMC811 and Al‐NaNMC811 cathodes tested in half cells at 1.5–4.1 V with a) 0.1 C, b) 0.2 C, and c) 0.5 C. d) Rate capabilities from 0.1 C to 5 C. Charge‐discharge profiles upon different cycles at 0.1 C for e) NaNMC811 and f) Al‐NaNMC811.

**Figure 7:**
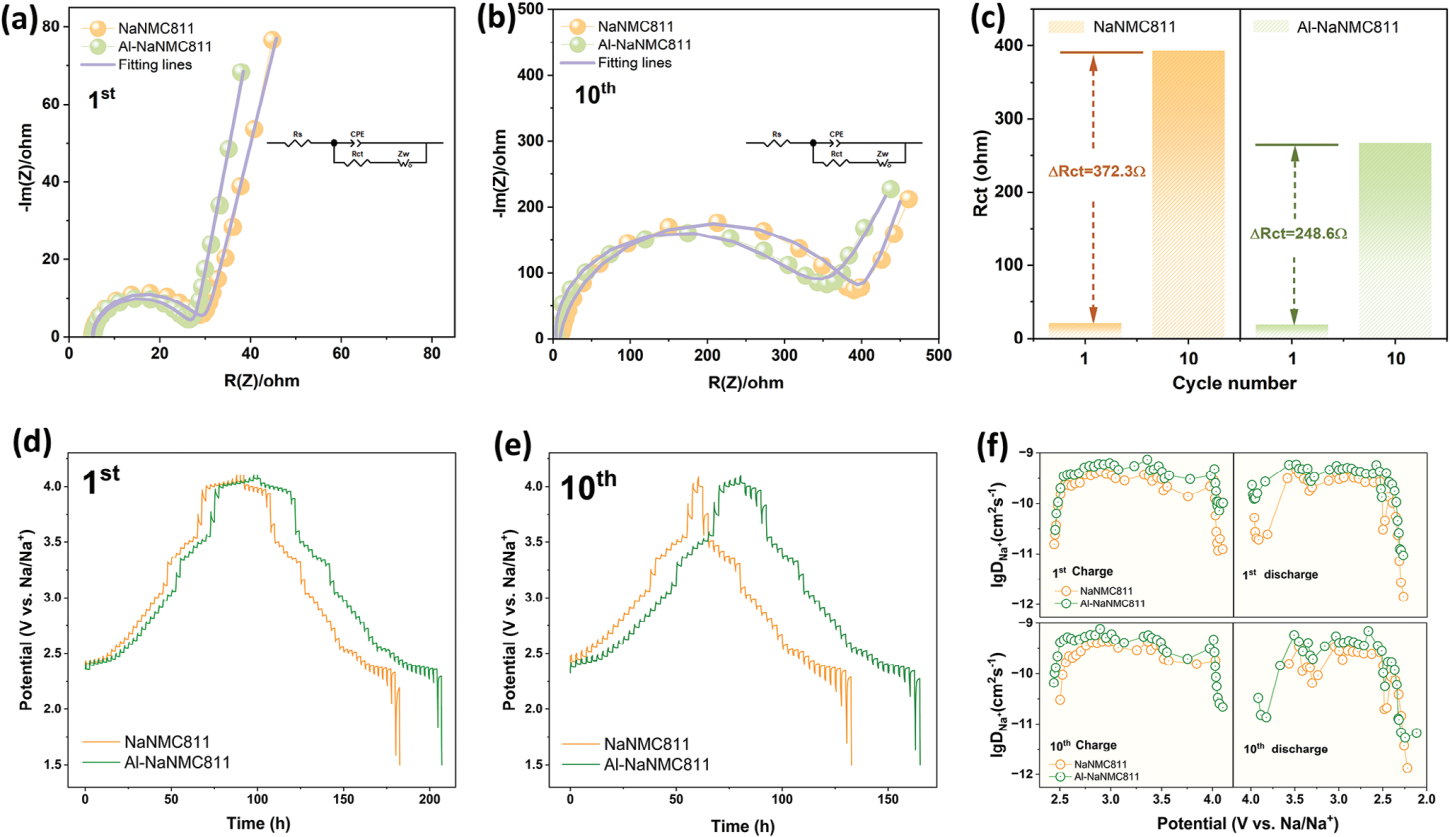
Electrochemical performance of NaNMC811 and Al‐NaNMC811. Nyquist plots of NaNMC811 and Al‐NaNMC811 measured under 80% SOC after a) the initial cycle, and b) the 10th cycle. c) *R*_ct_ values for the cathodes. GITT profiles for the charge/discharge process of NaNMC811 and Al‐NaNMC811 after d) the formatting cycle, and e) the 10th cycle. f) Diffusion coefficient of Na^+^ calculated from the GITT measurement.

**Figure 8:**
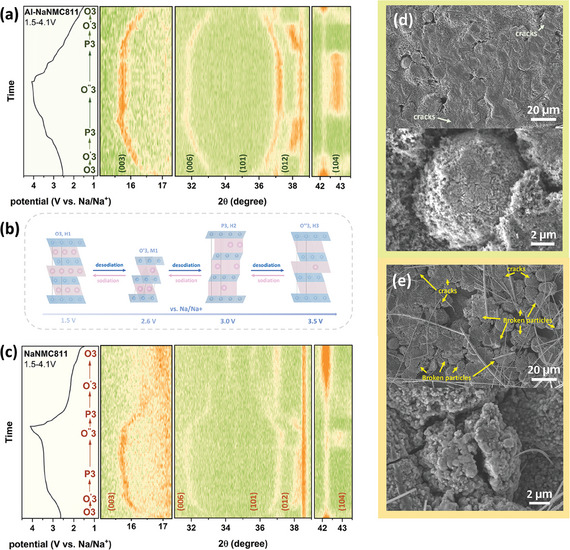
Initial charge‐discharge curves and contour maps of the *operando* XRD patterns under 0.1C, 1.5–4.1 V (vs Na/Na^+^) of a) Al‐NaNMC811 and c) NaNMC811. b) Schematic illustration of the phase evolution during the sodiation‐desodiation process. SEM images of d) Al‐NaNMC811 and e) NaNMC811 electrodes after 100 cycles (0.1 C, 1.5–4.1 V).

The scanning electron microscopy (SEM) images show that NaNMC811 and Al‐NaNMC811 exhibit a similar spherical morphology with dense nano‐sized flakes packed on the surface of the secondary particles (**Figure** **4**a,b). A uniform separation of each element is proven by energy dispersive spectroscopy (EDS) for both samples (Figure 4c,d). The chemical compositions of NaNMC811 and Al‐NaNMC811 are confirmed by inductively coupled plasma optic emission spectroscopy (ICP‐OES), as shown in Table S5 (Supporting Information). However, an unexpected contamination can be observed on some particles (Figure S4, Supporting Information), which is identified as Na_2_CO_3_ based on the X‐ray photoelectron spectroscopy (XPS) results discussed below. This contamination results from the migration of Na ions from the layered structure to the interface, and their subsequent reaction with carbon dioxide and water in the ambient air.^[^ ^49^ ^]^ Given the sensitive characteristics of the Ni‐rich NaNMC811 material, it is crucial to maintain the freshness of the samples during storage and handling in isolated atmosphere to obtain high‐purity samples.

The Rietveld refinement results of the XRD data demonstrate a well‐formed O3‐type structure without any impurities, which belongs to the $R\bar{3}m$ space group (Figure 4e–g).^[^ ^15^, ^16^, ^17^, ^18^, ^19^ ^]^ The lattice parameters of NaNMC811 are *a* (*b*) = 2.9466(9) Å and *c* = 15.7620(1) Å, while for Al‐NaNMC811 are *a* (*b*) = 2.9428(9) Å and *c* = 15.7813(1) Å, both in line with Hwang et al.'s work.^[^ ^15^ ^]^ The crystalline interplanar spacing values calculated from the refinement results are listed in Table S6 (Supporting Information). Additionally, a DFT study was carried out to depict Al‐gradient doping effect on Bader charge distribution within the NaNMC811 lattice as presented in Figure S5 (Supporting Information). After doping, the TMs adjacent to Al primarily exhibit electron gain as indicated by the positive charge transfer values in the figure, as Al has the lowest electronegativity compared to TM and O. Besides, the difference in electronegativity between Al and O is larger than that between TM and O, which may also contribute to the charge transfer and is plausibly one of the reasons for the Al–O bonding energy being higher than TM‐O, contributing to the overall structural stability.

To unveil the nanoscale structural information, transmission electron microscopy (TEM) is applied for the NaNMC811 and Al‐NaNMC811 cathode materials. As illustrated in **Figure** **5**a, a linear ordered lattice fringe is observed for NaNMC811, which can be well‐indexed to the O3‐type structure ($R\bar{3}m$). The measured crystalline interplanar spacing is 5.22 Å for (003) and 2.43 Å for (012), which are similar to the values calculated from the refinement XRD result (Figure 5b, l(003) = 5.25 Å, l(012) = 2.43 Å). Such distinct crystal structure is also observed in the TEM images of Al‐NaNMC811 (Figure 5c), where the (003) and (104) interplanar spacing are measured to be 5.29  and 2.14 Å, respectively. These are also in line with the calculated XRD results (Figure 5d, l(003) = 5.26 Å, l(104) = 2.14 Å). Both the TEM and XRD results demonstrate that Al‐NaNMC811 has a dilated interlayer spacing in the *c* orientation, and such directional expansion is expected to facilitate Na^+^ diffusion in the layered structure during the charge‐discharge processes.^[^ ^13^, ^30^, ^50^ ^]^ A low magnification TEM image of a lamella of each sample is shown in Figure S6 (Supporting Information).

To confirm the distribution of Al ions, the relative atomic ratio of Al to other elements (O, Na, Ni, Co, and Mn) is obtained by a line scan in the radial orientation of the Al‐NaNMC811 lamella using a high‐speed energy‐dispersive X‐ray (TEM‐EDX) measurement (Figure 5e). As illustrated in Figure 5f, the Al content significantly decreases from the surface to the interior of the lamella, indicating a gradient in the Al concentration within the surface layer of the Al‐NaNMC811 particles. The Al doping, decreasing radially from the surface towards the center of the particle, is observed to extend from the surface to about 0.1 µm towards the interior of the primary particle. This unique distribution of Al is expected to contribute to NaNMC811 structural stability and electrochemical performance improvement as discussed in the next section.

XPS measurement for the NaNMC811 and Al‐NaNMC811 cathode materials also confirms the Al‐gradient doping. The atomic concentrations are listed in Table S7 (Supporting Information), while the XPS measurements for Na, Ni, Co, and Mn are shown in Figure S7 (Supporting Information). In Figure 5g, the peaks in the C 1s spectra at 286.4 and 289.0 eV correspond to C–O and ${\mathrm{CO}}_{3}^{2-}$, respectively, indicating the existence of a Na_2_CO_3_ contamination.^[^ ^51^ ^]^ As discussed in context of the SEM analysis, impurities present on particle surfaces, resulting from reactions with atmospheric CO_2_ and moisture, are readily formed and frequently detected for NaNMC materials.^[^ ^49^ ^]^ The peak at 531 eV in the O 1s spectra (Figure 5h) confirms the ${\mathrm{CO}}_{3}^{2-}$ contribution while the lower energy peak at 528.3 eV corresponds to lattice oxygen.^[^ ^52^, ^53^, ^54^, ^55^ ^]^

The peak found in the Al 2p spectrum for the Al‐NaNMC811 sample confirms the enrichment of Al on the particle surface, with 1.9 at% compared to the average Al content of 0.18 at% (0.16 wt%) from ICP‐OES. In contrast, no peaks related to Al exist in the NaNMC811 sample spectrum (Figure 5i) as expected. The XPS results further validate the Al‐enriched surface of Al‐NaNMC811 and confirm the gradient doping from the perspective of Al existing on the surface.

### 2.3 Electrochemical Investigations

Electrochemical studies on NaNMC811 and Al‐NaNMC811 are performed in a SIB coin cell set‐up. **Figure** **6**a–c shows the cycling performance of NaNMC811 and Al‐NaNMC811 half cells tested at the voltage range of 1.5–4.1 V with current densities of 0.1 C, 0.2 C, and 0.5 C (1 C = 187 mAh g^−1^^[^ ^15^ ^]^). After 100 cycles, the capacity retentions reported for NaNMC811 are 57% at 0.1 C, 54% at 0.2 C, and 58% at 0.5 C. In contrast, Al‐NaNMC811 reports higher capacity retentions of 63% at 0.1 C, 60% at 0.2 C, and 62% at 0.5 C. The differences are plausibly attributed to the higher bond energy of Al–O which stabilizes the Al‐NaNMC811 crystal structure and lowers polarization, as observed from cyclic voltammetry measurements (Figure S8, Supporting Information).^[^ ^56^, ^57^ ^]^ For neither NaNMC811 nor Al‐NaNMC811, the varied current densities have no significant impact on capacity retention. The cell performances at high current density are also shown in Figure S9 (Supporting Information). The rate capability measurements were carried out from 0.1 C to 5 C in the same voltage range of 1.5–4.1 V. (Figure 6d). Al‐NaNMC811 (121/107 mAh g^−1^, 2/5 C) reports higher capacities than the NaNMC811 (94/78 mAh g^−1^, 2/5 C) under high‐rate discharging, reflecting faster kinetics of Na^+^ ions in Al‐NaNMC811. The improvement can be attributed to the enlarged (003) interplanar spacing (Table S6, Supporting Information). When the current density returns to 0.1 C, Al‐NaNMC811 (83%) shows higher capacity retention than NaNMC811 (74%), indicating an improvement in the stability of the layered‐oxide structure. The cell‐performance comparison to Hwang et al.'s work is listed in Table S8 (Supporting Information).^[^ ^15^ ^]^ Figure 6e,f shows the charge and discharge profiles at 0.1 C for NaNMC811 and Al‐NaNMC811. For the NaNMC811 cathode, a rapid capacity decrease (19% capacity loss) is observed during the first 10 cycles, which might be attributed to the instability of the pristine layered structure. On the other hand, this capacity decrease is alleviated in the Al‐NaNMC811 cathode which reports 14% capacity loss. dQ/dV graphs for NaNMC811 and Al‐NaNMC811 are shown in Figure S10 (Supporting Information). The electrochemical studies demonstrate that Al‐gradient doping is an effective strategy for enhancing the rate capability and capacity retention of NaNMC811.

To investigate the mechanism behind the cycling stability and rate capability enhancement, electrochemical impedance spectroscopy (EIS) and galvanostatic intermittent titration technique (GITT) were applied for NaNMC811 and Al‐NaNMC811 after the formatting and 10th charge–discharge cycle at 0.1 C. Generally, from the EIS measurement (**Figure** **7**a,b), a semicircle with a straight tail is observed in all Nyquist plots. The diameter of the semicircle represents the charge‐transfer resistance (R_ct_) while the left intercept of the semicircle with the real axis in the high‐frequency region is related to the electrolyte resistance (R_s_). The straight tail in the low‐frequency region represents the Warburg impedance related to the diffusion of Na^+^ (Z_w_).^[^ ^58^, ^59^ ^]^ An equivalent circuit shows excellent fitting results with the measurement data (Table S9, Supporting information). As shown in Figure 7c, R_ct_ increases drastically during the first 10 cycles. For NaNMC811, R_ct_ increases from 20.8 to 393.1 Ω after 10 cycles (Δ R_ct_ = 372.3 Ω). In contrast, for Al‐NaNMC811, R_ct_ increased from 18.7 to 267.3 Ω (Δ R_ct_ = 248.6 Ω). The smaller R_ct_ growth indicates that the Al doping improves the structural stability structure during the intercalation‐deintercalation processes, which plays a significant role in mitigating capacity reduction at the first 10 cycles.^[^ ^60^ ^]^

GITT method is utilized to study the Na^+^ diffusion in NaNMC811 and Al‐NaNMC811 depending on the degree of sodiation.^[^ ^61^, ^62^ ^]^ The diffusion coefficient values of Na^+^ ($D_{\mathrm{Na}}^{+}$, cm^2^ s^−1^) for both cathodes are calculated according to the GITT plots obtained after the initial and 10th cycle (cycled at 0.1 C, 1.5–4.1 V vs Na/Na^+^).^[^ ^63^ ^]^ As depicted in Figure 7f, Al‐NaNMC811 enables a higher $D_{\mathrm{Na}}^{+}$ (around 5.9× 10^−10^/1.2× 10^−11^ cm^2^ s^−1^) at each stage of the Na^+^ insertion‐extraction process for the initial and 10th cycles compared to pristine NaNMC811 (around 4.6× 10^—10^/1.4× 10^−12^ cm^2^ s^−1^). Figure 7d,e shows that Al‐gradient doping on NaNMC811 diminishes both the polarization and voltage decay over time (which is also observed in Figure 6e,f), demonstrating the improvement in the electrochemical kinetics during the sodiation–desodiation processes in Al‐NaNMC811.^[^ ^32^ ^]^

To further understand the structure evolution mechanism of the NaNMC811 and Al‐NaNMC811 during the charge‐discharge processes, *operando* XRD measurements were applied at 0.1 C between 1.5 and 4.1 V (vs. Na/Na^+^) at the initial cycle. The XRD contour maps of Al‐NaNMC811 during cycling are plotted in **Figure** **8**a. At the early stage of Na^+^ deintercalation (∼ 2.6 V vs Na/Na^+^), the (003) and (006) peaks gradually move to lower 2θ angle, revealing the expansion in the lattice parameter *c*. The larger plane spacing is caused by the increased electrostatic repulsion between neighboring oxygen planes surrounding Na^+^.^[^ ^64^ ^]^ Meanwhile, the (101) and (012) peaks shifting to higher 2θ angle mainly reflect the decrease in the lattice parameter *a*(*b*). Such variation in the interplanar distance suggests that the pristine O3‐type hexagonal phase ($R\bar{3}m$, H1) distorts into the O^'^3‐type monoclinic phase (*C2/m*, M1).^[^ ^65^ ^]^ As the potential sifted to higher values continually (2.6–3.0 V vs Na/Na^+^), the (003) and (006) peaks move to lower 2θ angles. Two peaks at higher 2θ angles eventually emerge after the (101) and (012) peaks vanish, demonstrating a complete phase transformation from an O^'^3‐type to P3‐type hexagonal phase (*R3m*, H2).^[^ ^66^, ^67^ ^]^ With further Na^+^ removal from the crystal structure (potential higher than 3.5 V vs Na/Na^+^), the formed P3 ‐ type phase starts converting into an O^''^3‐type hexagonal phase ($R\bar{3}m$, H3). This O^''^3‐type structure is similar to the initial O3 structure, however, the lattice parameter are different due to the lower Na^+^ content in the layered structure.^[^ ^67^ ^]^ During the P3 ‐ O^''^3 phase transition process, the (003) and (006) peaks slightly shift to higher 2θ angles, which persists until the end of charging at 4.1 V. Since during the discharge process inverse reactions compared to the charging occur, Na^+^ are inserted back into the crystal lattice and the structure reverts to the pristine O3‐type structure. A series of complex phase evolution (O3, H1 ‐ O^'^3, M1 ‐ P3, H2 ‐ O^''^3, H3 ‐ P3, H2 ‐ O^'^3, M1 ‐ O3, H1) for both materials is exhibited in Figure 8b. Throughout the entire charge‐discharge processes, the TM layer gliding mechanism plays an essential role in all the phase transitions, demonstrating high reversibility.^[^ ^67^, ^68^ ^]^

A contour map of the *operando* XRD patterns for NaNMC811 is shown in Figure 8c. Although the phase evolution is similar for both cathode materials, NaNMC811 shows faster O^'^3 ‐ P3 conversion during desodiation and O^''^3 ‐ O3 conversion during the sodiation process (Figure S11, Supporting Information), suggesting that the instability observed in the electrochemical measurements (Figure 6e) is caused by faster and more drastic structural changes compared to the Al‐NaNMC811 counterpart. In comparison, Al‐NaNMC811 shows more symmetrical and reversible phase transitions and has a generally smoother phase evolution, which helps to mitigate structural distortion brought about by the gliding of the TM layer.^[^ ^32^ ^]^ This gliding in Al‐NaNMC811 is alleviated by the higher bonding energy of Al–O compared to TM‐O so that Al–O sites function as pillars or an elastic band holding the different layers (O and TM) together during the structural changes induced by sodium (de)intercalation. Thus, they hinder drastic abrupt changes, which lead to irreversible dislocations.

This moderate structural evolution improves capacity retention (Figure 6a–c) as it contributes to the preservation of morphological characteristics of the secondary particles during the charge and discharge processes. After 100 cycles, Al‐NaNMC811 particles preserve their morphology with only a few microcracks observed, as shown in Figure 8d. Additionally, this gradient Al‐doping with an Al‐rich surface on the secondary particles might function as a protective layer similar to coating suppressing side reactions between the electrolyte and active material surface alongside with suppressing excessive structural changes. In contrast, the NaNMC811 particles exhibit notable cracks, with some particles destroyed into smaller fragments (Figure 8e). There is no obvious differences in morphology between the electrodes before cycling, which indicates that the particle cracking is due to electrochemical aging(Figure S12, Supporting Information). Therefore, the Al‐gradient doping is deemed to play a crucial role in maintaining structural and morphological stability during charge and discharge processes also at macroscopic level. The improved stability extending form the molecular level (*operando* XRD) to macroscopic explains the improvement in capacity retention of Al‐NaNMC811 during long‐term cycling (Figure 6a).

## 3 Conclusion

This work provides a comprehensive study of a pristine and Al‐gradient doped O3‐type NaNMC811 cathode material for SIBs. in situ HT‐XRD was utilized to investigate the phase transition mechanism during the NaNMC811 synthesis process. A clear $P\bar{3}m1$ ‐ $Fm\bar{3}m$ ‐ $R\bar{3}m$ phase evolution is identified, providing an understanding of the sodiation reactions during calcination. Furthermore, novel Al‐gradient doping was successfully implemented by coating the precursor with Al_2_O_3_ before sodiation. The electrochemical investigations report that Al‐NaNMC811 managed to sustain 100 mAh g^−1^ capacity after 100 cycles at 0.1 C (1.5 ‐ 4.1 V) and improved capacity retention (63%) compared to pristine NaNMC811 (57%). The improved cycling stability and minimal charge‐transfer resistance growth during cycling are attributed to higher Al‐O bond energy (compared to TM–O) in the crystal structure, leading to a smoother phase transition (less structure distortion) during the charge and discharge processes. Moreover, Al‐NaNMC811 demonstrates an impressive 107 mAh g^−1^ capacity at 5 C, which exhibits an 18% enhancement compared to NaNMC811. The rate capability improvement is attributed to the smaller charge‐transfer resistance and the enlarged (003) interplanar spacing which facilitates swifter Na^+^ diffusion kinetics.

Our study addresses the research gap of O3‐type NaNMC811 cathode materials, and contributes to a comprehensive understanding of the properties of Ni‐rich layered oxide cathodes. Our findings reveal gradient doping as a method to improve the structural and interfacial stability for future SIBs development.

## 4 Experimental Section

### NMC811OH Precursor Coating

A Picosun R100 atomic layer deposition (ALD) reactor modified for powder coating and equipped with a POCA 3 powder reaction chamber was used for the NMC811OH precursor coating.

NMC811OH precursor powder (6 g) was evenly distributed in the POCA 3 powder chamber, which was sealed with a low‐porosity stopper to ensure secure containment and prevent significant flow restriction. The chamber was then installed inside the reactor and connected to two precursor vessels containing water and trimethylaluminium (TMA), which were kept at room temperature. The reactor was evacuated to achieve the necessary vacuum conditions, and the deposition process was conducted at 200 °C (±10 °C), with a 30 min stabilization period, a line flow rate of 30 sccm per source line (four lines total), an intermediate space flow rate of 120 sccm, and 18 ALD cycles. Each ALD cycle followed a precise sequence: 2×(TMA pulse → short purge) → TMA pulse → long purge → 2×(water pulse → short purge) → water pulse → long purge, with step timings of 0.1 s for pulses, 1 s for short purges, and 60 s for long purges. Upon completion of the deposition cycles, the reactor was cooled to 50 °C to prevent material decomposition, and the coated powder was transferred to a glass vial and stored in an Ar‐filled glovebox.

### Synthesis of NaNMC811 and Al‐NaNMC811

An industrial precursor of NMC811OH (Umicore Battery Materials Finland, Finland) and its Al_2_O_3_ coating product was sufficiently mixed with NaOH (NaOH: NMC811OH = 1.01:1 molar ratio) by agate mortar inside an Ar‐filled glovebox (MO‐5, VAC, America) to avoid the hydrolysis of NaOH (⩾98%, Sigma‐Aldrich, America). Both mixtures were calcined inside a tube furnace (Nabertherm R70/9, Germany) at 625 °C for 24 h with oxygen flow with a heating rate of 3 °C min^−1^ and cooling rate of 1.5 °C min^−1^. After calcination, the samples were stored in a glovebox.

### Material Characterization

Phase transition analysis of the NaNMC811 synthesis process was performed by in situ high‐temperature X‐ray diffraction (in situ HT‐XRD, Cu Kα1, PANanalytical, UK) with a detachable high‐temperature oven (HTK 1200N, Anton Paar, Australia) in the range of 10°‐ 85° under air condition. The oven was heated 100 °C to 800 °C with a 2°*C* min^−1^ heating rate. Each XRD pattern was measured at 20 °C interval below 600 °C and 50 °C interval above 650 °C for 30 min. Under 625°*C*, the sample was held for 24 h. During the cooling process (2 °C min^−1^ cooling rate), each XRD pattern was measured at 50 °C interval. NaNMC811 and Al‐NaNMC811, the crystalline phases were examined by powder X‐ray diffraction (XRD, Cu Kα1 1.5046 Å45 kV 40 mA, PANanalytical, UK) in the range of 10°–85° at 2.5° min^−1^ under room temperature. The detailed crystal structure, lattice parameters, and phase fraction were analyzed by the Rietveld method via the program FULLPROF software. The VESTA software was used to plot the crystal model and calculate the crystalline interplanar spacing.

A mixture (15 mg) of NaOH and the Ni_0.8_Mn_0.1_Co_0.1_(OH)_2_ (1.01:1 molar ratio) precursor was prepared. for thermogravimetric (TG) and differential scanning calorimetric (DSC) experiments measured on a thermogravimetric analysis (STA 449 F3, NETZSCH, Germany) with a heating rate of 10K min^−1^ form 40 °C to 900 °C under air.

The morphologies of the NaNMC811 and Al‐NaNMC811 powders were observed using scanning electron microscopy (SEM, JEOL‐4700 F, Japan) with energy‐dispersive X‐ray spectroscopy (EDS) analysis. Micro‐structures were characterized by transmission electron microscopes (TEM, JEOL JEM‐2800, Japan) with high‐speed energy‐dispersive X‐ray (EDX) analysis. For TEM sample preparation, cross‐sectional TEM lamellas were prepared by focused ion beam (FIB) on a JEOL JIB‐4700F microscope. To avoid Ga ion beam damage particles, a thickness of 1.2 µm Pt protective layer was deposited on the top of the interest secondary particle before cutting and milling. For coarse milling and fine polishing, the Ga ion beam energy/probe current with 30 kV/10 nA and 5 kV/30 pA was applied, respectively. The thickness of the final TEM lamellas is about 100 nm for the HR‐TEM analysis. An inductively coupled plasma optic emission spectroscopy (ICP‐OES, Agilent 5900 SVDV, America) instrument was used to analyze the elemental composition of the synthesized and doped samples.

Spin‐polarized density functional theory calculation was applied using the Vienna Abinitio Simulation Package (VASP)^[^ ^69^ ^]^ codes with the help of VASPKIT.^[^ ^70^ ^]^ The projector‐augmented wave (PAW) method was used to model the core‐valence electron interactions.^[^ ^71^ ^]^ The PerdewBurke‐Ernzerhof (PBE) functional of the generalized gradient approximation (GGA) was used in the calculations,^[^ ^72^ ^]^ jointly with an energy cutoff of 500 eV. The Gaussian smearing method was used to determine the partial occupancies of the orbitals with the smearing width of 0.05 eV. The DFT‐D3 method of Grimme with zero‐damping function was adopted to account for the van der Waals correction.^[^ ^73^ ^]^ The DFT + U method was employed in the calculations, with the effective U values of 6.4 eV for 3d orbital of Ni.^[^ ^74^ ^]^ The convergence accuracies of energy and force were set to be 10^−5^ eV and 0.03 eV Å^−1^, respectively. A Monkhorst–Pack grid with a k‐point density of 0.03 Å^−1^ was used for Brillouin zone sampling. For the calculation model before Al doping, the simplified NaNMC811 ($R\bar{3}m$) unit cell was used to simplify the computations. After expanding this unit cell to a 3 × 3 × 1 supercell, one Al atom was substituted for Ni, which served as the model for Al doping calculations.

X‐ray photoelectron spectroscopy (XPS) for the NaNMC811 and Al‐NaNMC811 powder was carried out with a Kratos Axis Ultra spectrometer (Kratos Analytical, Japan) with monochromated Al Kα‐radiation, pass energy of 40 eV, an X‐ray power of 150 W and an analysis area of approximately 700 µm × 300 µm. The binding energy scale is based on instrument calibration and no additional binding energy correction was applied. The elemental composition was determined from peak areas of high‐resolution core‐level spectra after Shirley background subtraction using equipment‐specific sensitivity factors.

### Electrochemical Measurements

For both NaNMC811 and Al‐NaNMC811 cathodes, active material: carbon black (Super C65, Timcal, Switzerland): polyvinylidene fluoride (PVDF, Sole 5130, Solvay, Belgium) = 85: 10: 5 mass ratio was used to prepare the slurry. The resulting slurry was applied to an aluminum foil and first dried inside an Ar‐filled chamber overnight. Afterward, the cathodes were transferred into a vacuum oven (VTR 5022, Heraeus, Germany) for drying under 80 °C for 5 h. The mass loading of the cathode active material was 2.0–2.3 mg cm^−2^.

CR2016‐type coin cells (Hohsen, Japan) were fabricated in half‐cell configurations inside an Ar‐filled glovebox (<1 ppm of H_2_O and O_2_, Jacomex, France). The half‐cell was composed of a 14 mm diameter cathode (NaNMC811 and Al‐NaNMC811), a sodium‐metal foil as a counter electrode, and glass fiber (Whatman GF/A) separator soaked in sodium hexafluorophosphate solution as an electrolyte (1 M NaPF_6_, E‐Lyte, Germany). Three‐electrode cells (ECC‐ref, EL‐CELL, Germany) were assembled using a cathode, sodium–metal foil as the counter and reference electrodes, and a glass fiber separator soaked in 1 M NaPF_6_ (200 µL) solution.

Electrochemical evaluations of half‐cells at ambient temperature were recorded with a Land test system (CT2001A, LANHE, China). Electrochemical impedance spectroscopy (EIS), galvanostatic intermittent titration technique (GITT), and cyclic voltammetry (CV) were measured by the three‐electrode cells on a potentiostat (MPG‐205, Biologic, France). EIS measurements were carried out at 80% state‐of‐charge (SOC) during the discharge process over the frequency range of 20 kHz and 1 mHz using an alternating potential perturbation amplitude of 10 mV after the initial and 10th cycles at 0.1 C. The equivalent circuit fitting of EIS is applied by Zview software. GITT was measured at 30 min 0.05 C current pulse and 2 h relaxation in the potential range of 1.5 ‐ 4.1 V (vs Na/Na^+^) after the initial and 10th cycles at 0.1 C. CV measurements were performed between 1.5 and 4.1 V (vs Na/Na^+^) at 0.2 mV s^−1^.

*Operando* X‐ray diffraction (*operando* XRD) analysis was conducted to investigate the mechanism of different rate capabilities for NaNMC811 and Al‐NaNMC811. An ECC‐Opto‐Std test cell (EL‐CELL, Germany) equipped with a beryllium window for X‐ray transmission was employed. Charging and discharging in the Opto cell were measured at 0.1 C within the voltage range of 1.5 – 4.1 V (vs Na/Na^+^) using an Ivium Vertex Potentiostat (Ivium, Netherlands). XRD diffraction patterns were recorded within the 2θ range of 15° to 46°.

## Conflict of Interest

The authors declare no conflict of interest.

## Supporting information

Supporting Information

## Data Availability

The data that support the findings of this study are available from the corresponding author upon reasonable request.
