# Supplementary material for: Understanding of a Ni‐Rich O3‐Layered Cathode for Sodium‐Ion Batteries: Synthesis Mechanism and Al‐Gradient Doping
Source: Small. 2024 Nov 8;21(2):2408072. doi: 10.1002/smll.202408072 (PMC11735899; doi:10.1002/smll.202408072)
Supplement: Supplementary file 1 — Supporting Information [file SMLL-21-2408072-s001.pdf]

NANO · MICRO
small

Supporting Information

for *Small*, DOI 10.1002/smll.202408072

Understanding of a Ni-Rich O3-Layered Cathode for Sodium-Ion Batteries: Synthesis Mechanism and Al-Gradient Doping

Binglu Wang, Xiangze Kong, Filipp Obrezkov, Princess Stephanie Llanos, Jani Sainio, Alisa R. Bogdanova, Anna Kobets, Timo Kankaanpää and Tanja Kallio**

Supporting Information

Understanding of a Ni-rich O3-layered Cathode for Sodium-ion Batteries: Synthesis Mechanism and Al-gradient Doping

Binglu Wang, Xiangze Kong*, Filipp Obrezkov, Princess Stephanie Llanos, Jani Sainio, Alisa R. Bogdanova, Anna Kobets, Timo Kankaanpää, Tanja Kallio*

B. Wang, Dr. X. Kong, Dr. F. Obrezkov, P. Llanos, A. Bogdanova, A. Kobets, Prof. T. Kallio

Address: Department of Chemistry and Materials Science, School of Chemical Engineering, Aalto University, P.O. Box 16100, FI-00076 Aalto, Finland

Dr. J. Sainio

Department of Applied Physics, School of Science, Aalto University, P.O. Box 15100, FI-00076 Aalto, Finland

Dr. T. Kankaanpää

Address: Umicore Battery Materials Finland, 67101 Kokkola, Finland

Corresponding author email address: xiangze.kong@aalto.fi, tanja.kallio@aalto.fi

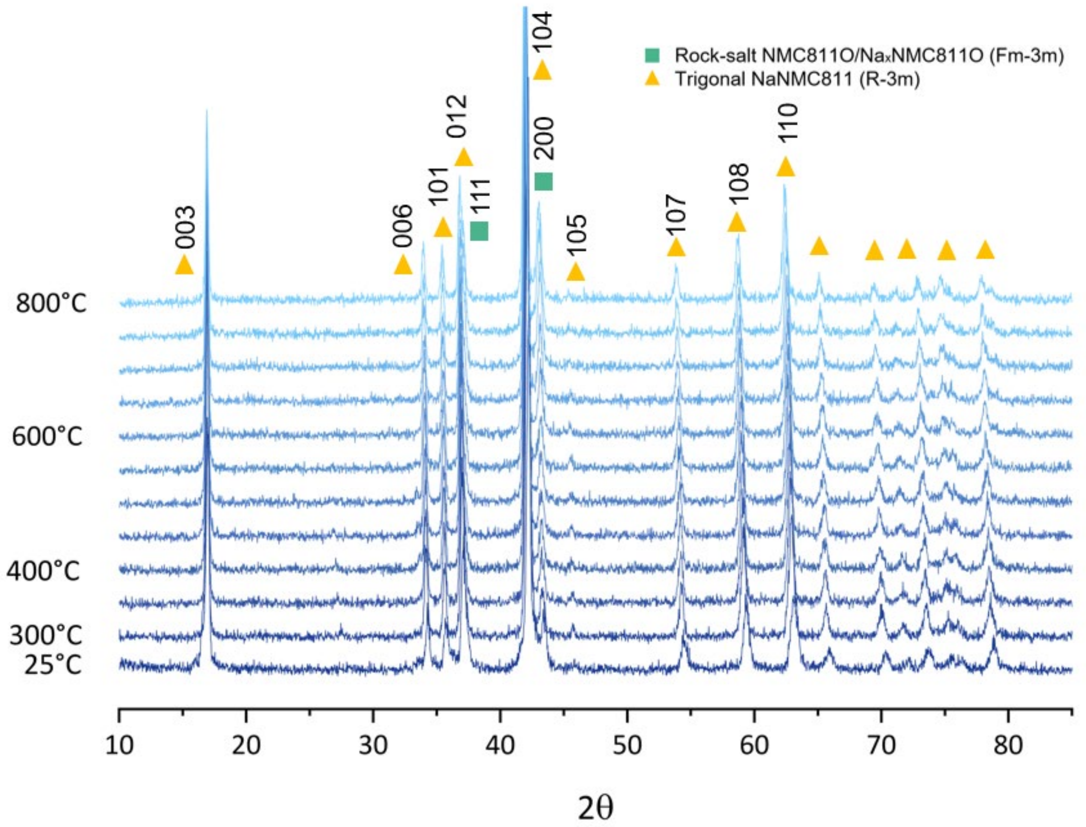

Figure S1. HT-XRD patterns recording for the cooling process from 800ºC to 25ºC.

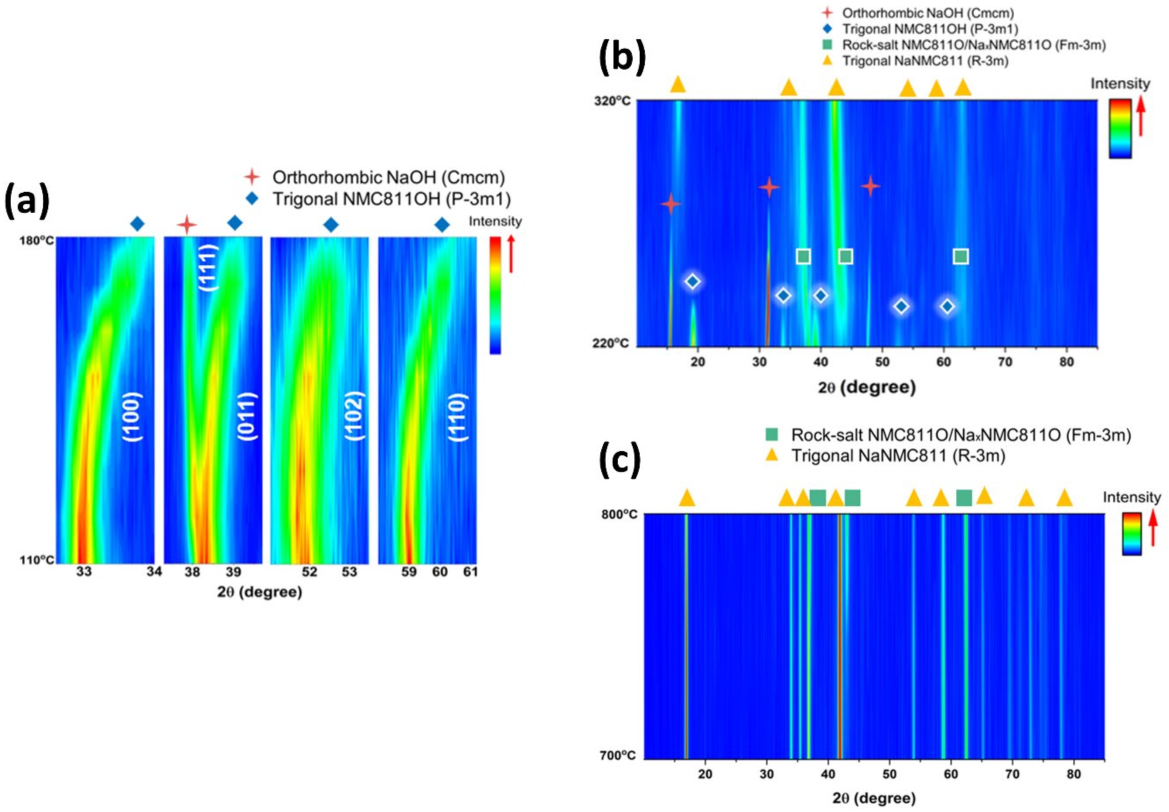

Figure S2. HT-XRD contour maps during the heating process: (a) 110°C - 180°C, (b) 220°C - 320°C, and (c) 700°C - 800°C.

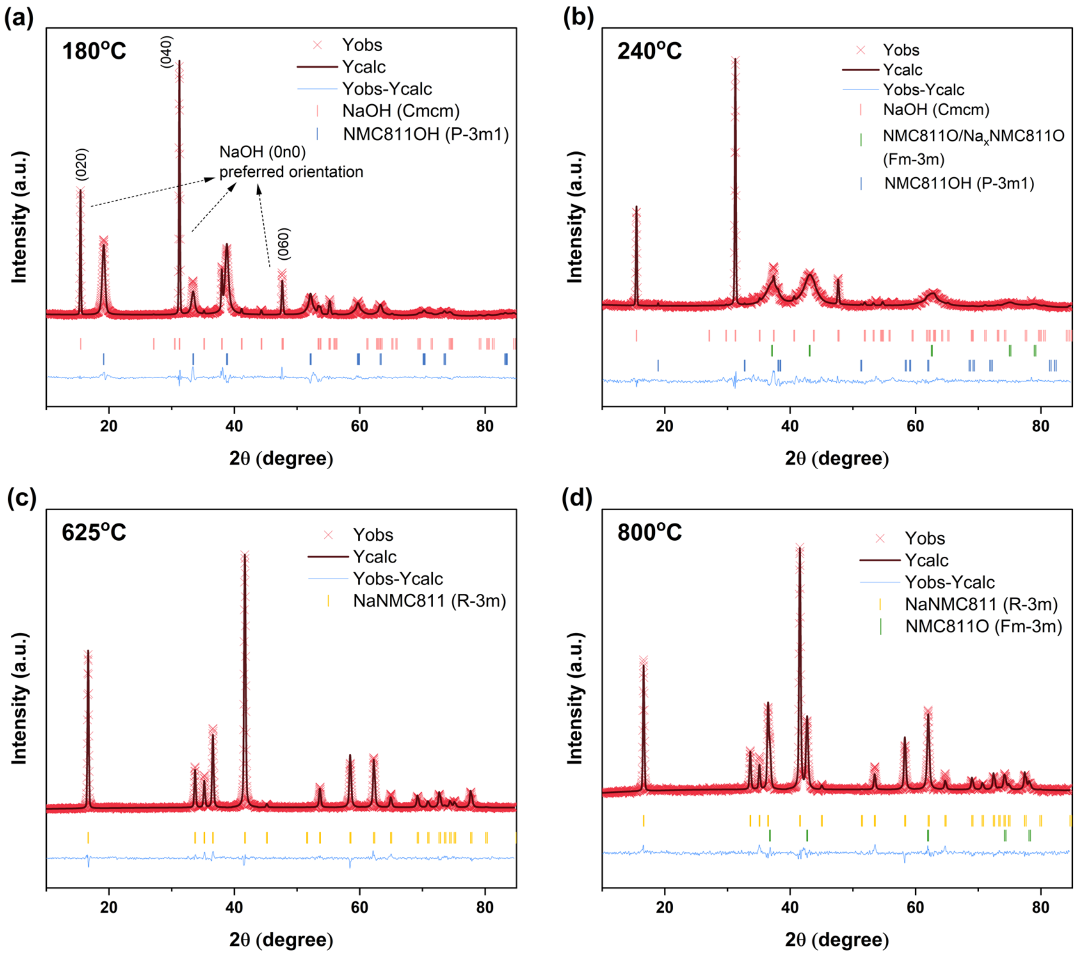

Figure S3. HT-XRD Rietveld refinement plots under typical temperatures of (a) 180°C, (b) 240°C, (c) 625°C, and (d) 800°C for the calculated NaNMC811 sample.

Table S1. HT-XRD Rietveld refinement results of the lattice parameters at 180ºC.

Compounds	Space group	Phase percentage (wt%)	Lattice parameters					
			a (Å)	*b* (Å)	*c* (Å)	*α* (∘)	*β* (∘)	*γ* (∘)
**NaOH**	*Cmcm*	30.3	3.4320(6)	11.4508(6)	3.4130(1)	90	90	90
**NMCOH811**	*P-3m1*	69.6	3.0971(2)	3.0971(2)	4.6369(9)	90	90	120

Table S2. HT-XRD Rietveld refinement results of the lattice parameters at 240ºC.

Compounds	Space group	Phase percentage (wt%)	Lattice parameters					
			a (Å)	*b* (Å)	*c* (Å)	*α* (∘)	*β* (∘)	*γ* (∘)
**NaOH**	*Cmcm*	6.1	3.4387(4)	11.4467(2)	3.4130(1)	90	90	90
**NMCOH811**	*P-3m1*	0.6	3.1593(1)	3.1593(1)	4.6878(9)	90	90	120
**NMC811O/ Na_xNMC811O**	*Fm-3m*	93.3	4.1978(7)	4.1978(7)	4.1978(7)	90	90	90

Table S3. HT-XRD Rietveld refinement results of the lattice parameters at 625ºC.

Compounds	Space group	Phase percentage (wt%)	Lattice parameters					
			a (Å)	*b* (Å)	*c* (Å)	*α* (∘)	*β* (∘)	*γ* (∘)
**NaNMC811**	*R-3m*	100	2.9820(9)	2.9820(9)	15.9639(1)	90	90	120

Table S4. HT-XRD Rietveld refinement results of the lattice parameters at 800ºC.

Compounds	Space group	Phase percentage (wt%)	Lattice parameters					
			a (Å)	*b* (Å)	*c* (Å)	*α* (∘)	*β* (∘)	*γ* (∘)
**NaNMC811**	*R-3m*	77.5	2.9907(5)	2.9907(5)	15.9897(8)	90	90	120
**NMC811O**	*Fm-3m*	22.5	4.2353(8)	4.2353(8)	4.2353(8)	90	90	90

Tables S1-S4 show detailed information on the lattice parameter evolution during the sodiation at four representative temperatures. As shown in Table S1, at 180ºC, the starting materials NaOH and NMC811OH exist. The very intense peaks ascribed to the (0*n*0) crystalline plane (n = 2, 4, 6) of NaOH result from the preferred orientation. During the experimental process, NaOH was ground (milled in a mortal with a pestle) from the tablets into powder, which could lead to the preferential orientation. Table S2 illustrates the existence of NaOH, NMC811OH, and NMC811O/Na_xNMC811O. At this temperature, the majority

of NMC811OH loses H/O from its original structure, thus only 0.6 wt-% of NMC811OH remains. Meanwhile, as sodium insertion into the layered structure proceeds, NaOH has fraction has also decreased (6.1 wt-%). As the temperature increases further, only pure NaNMC811 exists (Table S3). However, as shown in Table S4, the impurity rock-salt phase, plausibly NaNMC811O, appears again.

Table S5. Stoichiometric elemental indices based on the ICP-OES measurements.

**Sample**	**Elements**					**Formula**
	Na	Ni	Mn	Co	Al	
**NaNMC811**	0.96	0.80	0.096	0.096	0	$Na_{0.96}Ni_{0.80}Mn_{0.096}Co_{0.096}O_2$
**Al-NaNMC811**	0.99	0.80	0.099	0.099	0.007	$Na_{0.99}Ni_{0.80}Mn_{0.099}Co_{0.099}Al_{0.007}O_2$

The weight percentage of Al dopant for NaNMC811: 0.16 %.

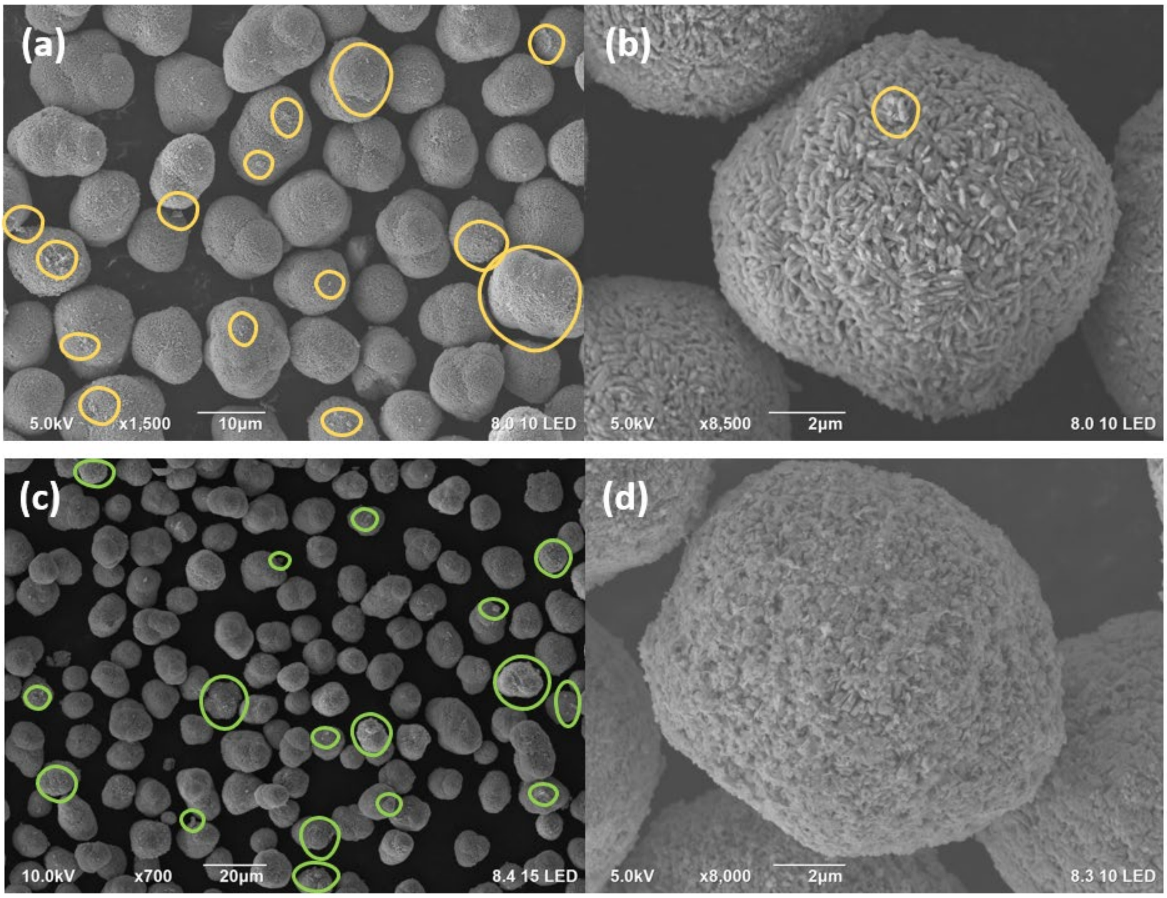

Figure S4. Contamination clusters observation on the particles of (a), (b) NaNMC811 and (c), (d) Al-NaNMC811.

Table S6. Crystalline inter-planar spacing of NaNMC811 and Al-NaNMC811 calculated from the Rietveld XRD refinement and the VESTA software.

**Crystalline plan**	**NaNMC811 plan distance (nm)**	**Al-NaNMC811 plan distance (nm)**
**(003)**	0.525	0.526
**(006)**	0.263	0.263
**(101)**	0.252	0.252
**(012)**	0.243	0.243
**(104)**	0.214	0.214
**(105)**	0.198	0.198
**(107)**	0.169	0.169
**(108)**	0.156	0.156
**(110)**	0.147	0.147

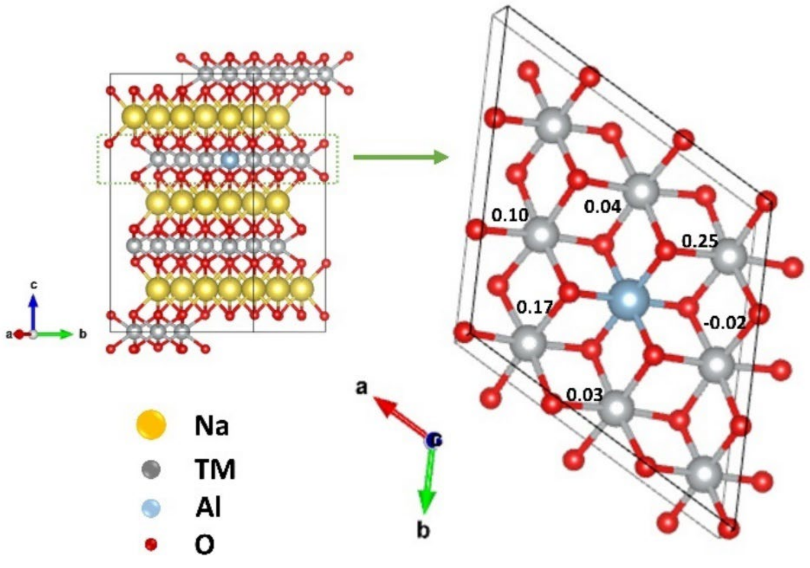

Figure S5. Bader charge analysis of simplified Al-NaNMC811.

DFT is utilized for bare $NaNiO_2$ and Al (1 at%) doped $NaNiO_2$ as simplified model compounds. $NaNiO_2$ has the same crystal structure as NaNMC811, both O3 and R-3m in the space group. As the Ni content in NaNMC811 is high, omitting Co and Mn in the structure is considered as a reasonable simplification for studying Al doping.
The electronegativity of O, Ni and Al decrease in the order O (3.5) > Ni (1.9) > Al (1.5). Consequently, Al tends to donate electrons more readily, when compared to Ni, and forms stronger bonds with O. This is expected to be beneficial by improving overall crystal structure stability, which is consistent with the fact that the Al-O (502 kJ mol^{-1}) bonding energy is higher when compared to Ni-O (336 kJ mol^{-1}).[S1]
Figure S5 illustrates the charge transfer occurring in the $NaNO_2$ structure after Al doping. Since Al has relatively low electronegativity (1.5), it tends to lose electrons more readily, which aligns with the observation that Ni adjacent to Al predominantly gains electrons.

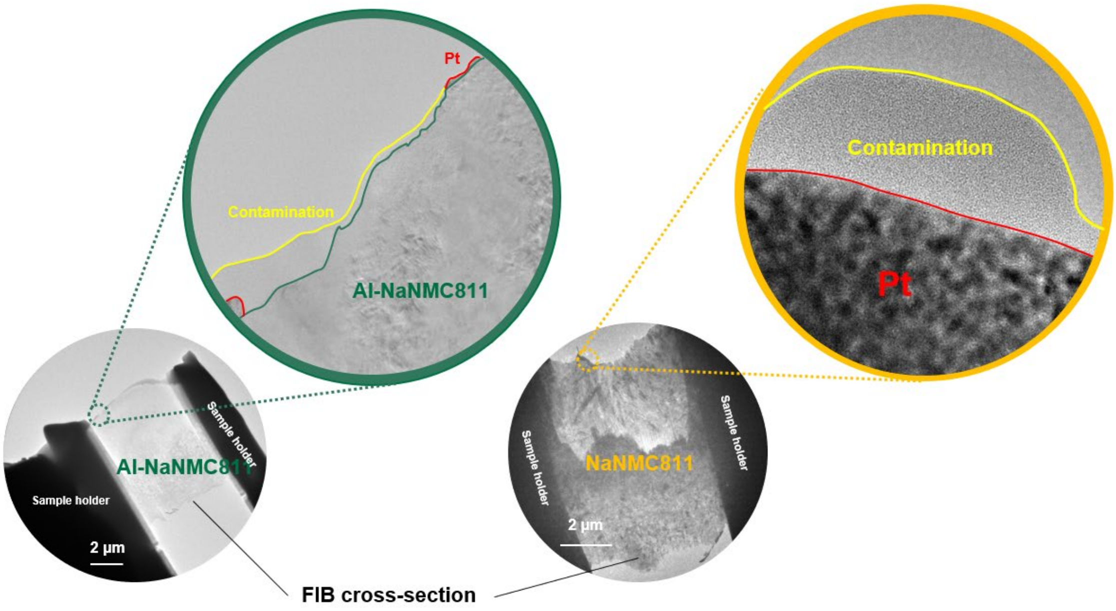

Figure S6. TEM lamella images of Al-NaNMC811 and NaNMC811.

Figure S6 shows the TEM lamella of NaNMC811 and Al-NaNMC811 fixed on sample holders. A Pt protective layer was deposited on the top of the secondary particles of interest to avoid Ga ion beam damage during lamella preparation (milling steps). Nano-scale contamination layers that might contain H/C are observed for both TEM lamella samples.

Table S7. Atomic concentrations on the surface of NaNMC811 and Al-NaNMC811 from XPS.

element	NaNMC811 (%)	Al-NaNMC811 (%)
**C**	31.9	26.5
**O**	49.1	45.4
**Na**	17.3	23.7
**Ni**	1.6	2.3
**Co**	0.1	0.2
**Mn**	-	-
**Al**	-	1.9

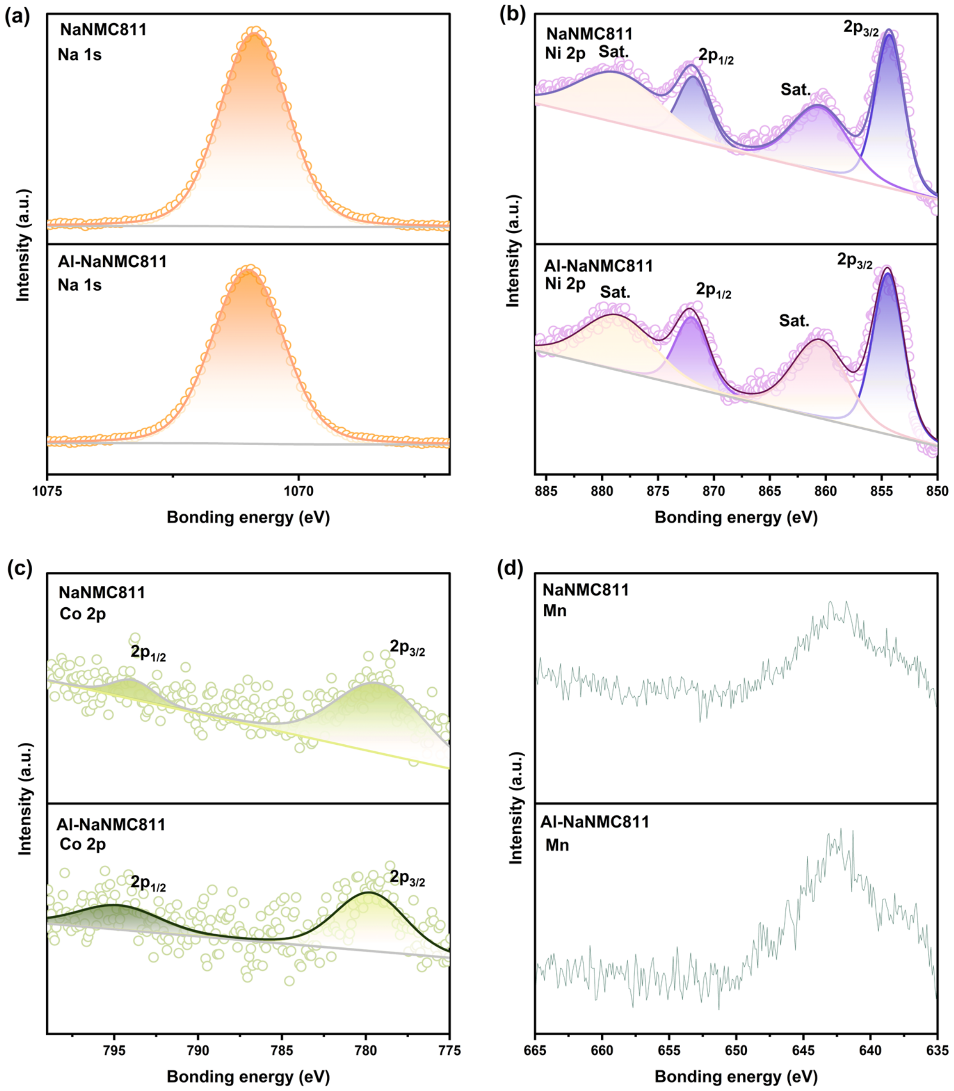

Figure S7. A sequence of the XPS surface profile for NaNMC811 and Al-NaNMC811 of (a) Na, (b) Ni, (c) Co, and (d) single of Mn overlaps with a Ni Auger peak.

Figure S7a shows the intense Na 1s peak at 1071.0 eV. Ni 2p spectra in Figure S7b exhibits peaks at 854.3 eV and 872.0 eV assigned to Ni $2p_{3/2}$ and Ni $2p_{1/2}$ with satellite peaks, respectively. Similarly, the Co 2p spectra are also identified in Figure S7c. However, the signals from Mn are extremely weak and overlap with a Ni Auger peak, which renders them difficult to discriminate (Figure S7d).

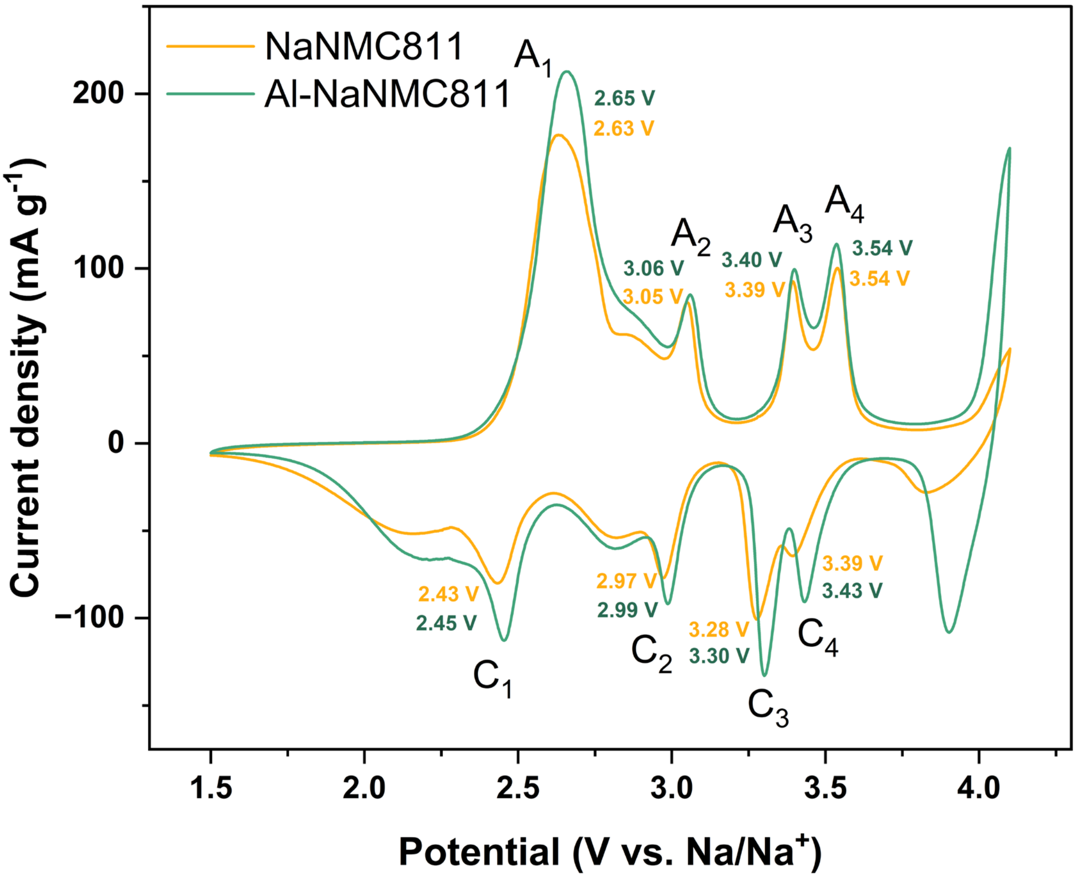

Figure S8. Cyclic voltammetry curves of the NaNMC811 and Al-NaNMC811 cathodes at a scan rate of 0.2 mV s^{-1}.

CV curves shown in Figure S8 of NaNMC811 and Al-NaNMC811 contain four main redox peak couples, which are assigned as C_1/A_1, C_2/A_2, C_3/A_3, and C_4/A_4 respectively. The electrochemical reversibility and the degree of potential polarization and be identified from the potential difference of each redox peak (ΔV).[S2][S3] For NaNMC811, $\Delta V(C_1/A_1)$ = 0.02 V, $\Delta V(C_2/A_2)$ = 0.08 V, $\Delta V(C_3/A_3)$ = 0.11 V, $\Delta V(C_1/A_1)$ = 0.15 V. For Al-NaNMC811, $\Delta V(C_1/A_1)$ = 0.02 V, $\Delta V(C_2/A_2)$ = 0.07 V, $\Delta V(C_3/A_3)$ = 0.10 V, $\Delta V(C_1/A_1)$ = 0.11 V.

The smaller potential differences of Al-NaNMC811 compared to NaNMC811 demonstrate the improved reversibility of the electrochemical reactions and the decreased polarization.

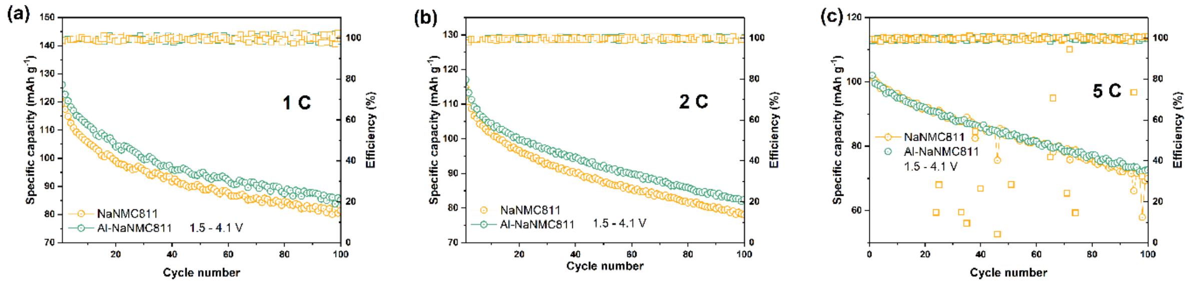

Figure S9. The rate capability experiments for NaNMC811 and Al-NaNMC811 based cathodes cycled at the potential range of 1.5 – 4.1 V in the half cells under high current densities of (a) 1C, (b) 2 C, and (c) 5 C.

Table S8. Comparison of the cycling performance of NaNMC811 and Al-NaNMC811 with the previous study.

materials	Voltage range (V vs. Na/Na^+)	Rate capacity (Normalized)	Cycle condition	Capacity retention	Ref.
**NaNMC811**	1.5 – 4.1	46% /5C	0.5C	58.0%/100 cycles	This work
**Al-NaNMC811**	1.5 – 4.1	55.7%/5C	0.5C	61.7%/100 cycles	This work
**NaNMC811**	1.5 – 4.1	55%/750 mA g^{-1}(~4C)	75 mA g^{-1} (~0.4C)	50%/100 cycles	[S4]

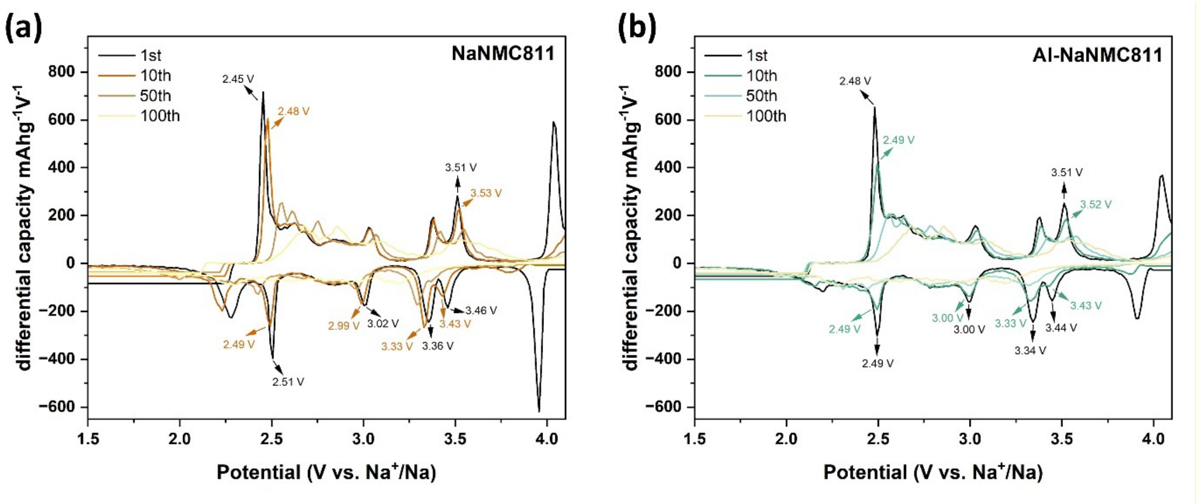

Figure S10. dQ/dV curves for (a) NaNMC811 and (b) Al-NaNMC811 (cycled in half cells at 0.1 C, 1.5 – 4.1 V).

Figure S10 shows the 1st, 10th, 50th, and 100th dQ/dV profiles of NaNMC811 and Al-NaNMC811. As the number of charge and discharge cycles increases, the redox peak potential in the dQ/dV profiles shifts toward more positive potentials for the anodic currents and towards more negative potentials for the cathodic currents indicating irreversible phase changes occur. Al-NaNMC811 has smaller potential changes between the 1st to 10th cycle for each redox peak compared to NaNMC811, which demonstrates a more stable structure after Al gradient doping. Additionally, the redox peaks located around 4.0 V in case of the NaNMC811 cathode undergo a sharp decline after the 10th cycle, resulting in a drastic capacity decrease.

Table S9. The fitting result of the EIS spectra.

	**After 1st cycle**				**After 10th cycle**			
	R_s (ohm)	Error (%)	R_{ct} (ohm)	Error (%)	R_s (ohm)	Error (%)	R_{ct} (ohm)	Error (%)
**Al-NaNMC811**	5.3	0.24	18.7	0.66	8.8	0.88	267.3	1.1
**NaNMC811**	5.2	0.53	20.8	0.47	8.9	0.97	393.1	0.76

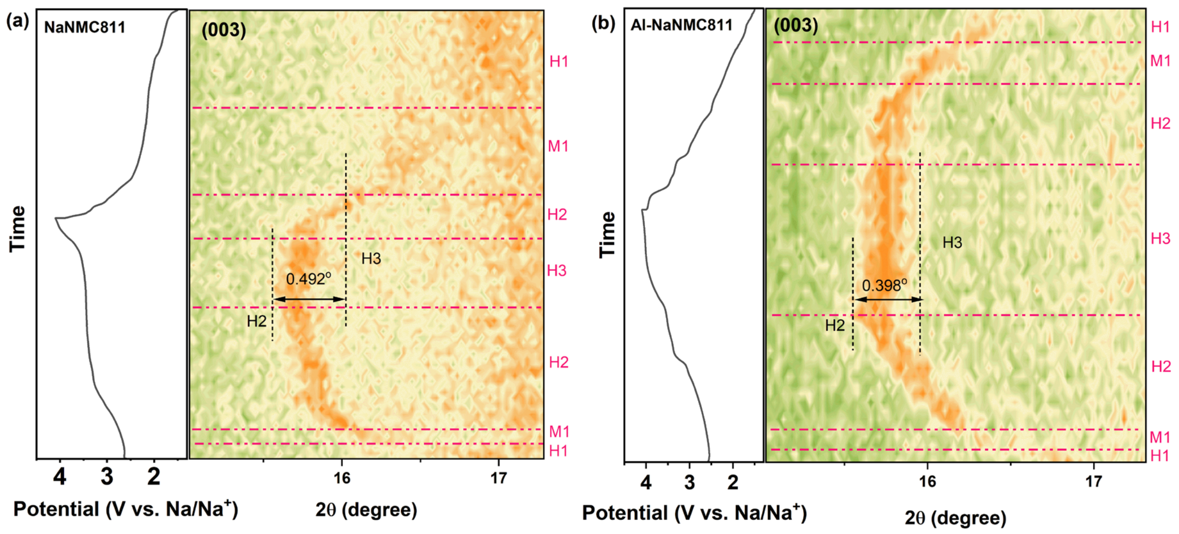

Figure S11. *Operando* XRD characterization during the charge/discharge process (1.5 – 4.1 V vs. Na/Na^+) for the (003) peak of (a) NaNMC811 and (b) Al-NaNMC811.

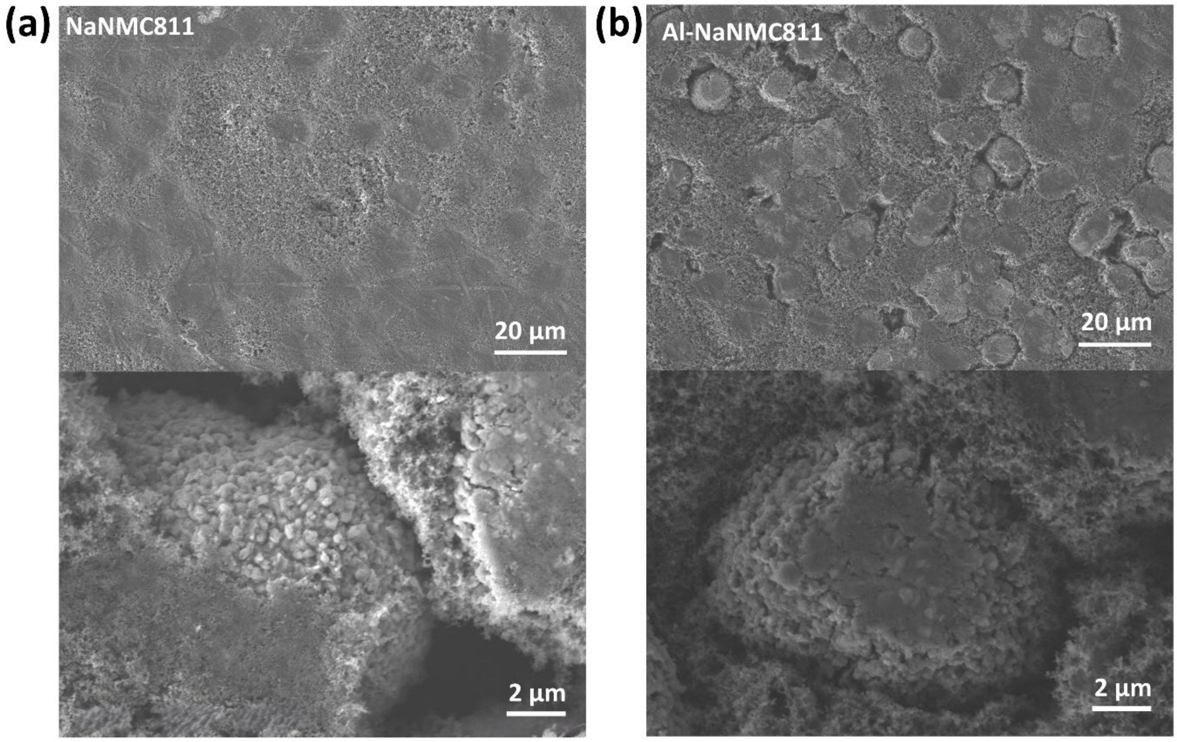

Figure S12. SEM images for the electrodes before cycling. (a) NaNMC811 and (b) Al-NaNMC811.

Reference

[S1] Pei, Ben, et al. "Al substitution for Mn during Co-precipitation boosts the electrochemical performance of $LiNi_{0.8}Mn_{0.1}Co_{0.1}O_2$." *Journal of the Electrochemical Society* 168.5 (2021): 050532.

[S2] Wang, Yang-Yang, et al. "Na-doped $LiNi_{0.8}Co_{0.15}Al_{0.05}O_2$ with excellent stability of both capacity and potential as cathode materials for Li-ion batteries." *ACS Applied Energy Materials* 1.8 (2018): 3881-3889.

[S3] Jeong, Mihee, et al. "Stabilizing effects of Al-doping on Ni-rich $LiNi_{0.80}Co_{0.15}Mn_{0.05}O_2$ cathode for Li rechargeable batteries." *Journal of Power Sources* 474 (2020): 228592.

[S4] Hwang, Jang-Yeon, et al. "A comprehensive study of the role of transition metals in O3-type layered $Na[Ni_xCo_yMn_z]O_2$ (x= 1/3, 0.5, 0.6, and 0.8) cathodes for sodium-ion batteries." *Journal of Materials Chemistry A* 4.46 (2016): 17952-17959.
